# Measuring Bone Healing: Parameters and Scores in Comparison

**DOI:** 10.3390/bioengineering10091011

**Published:** 2023-08-26

**Authors:** Nicolas Söhling, Olivia Von Jan, Maren Janko, Christoph Nau, Ulrike Ritz, Ingo Marzi, Dirk Henrich, René D. Verboket

**Affiliations:** 1 Department of Trauma, Hand and Reconstructive Surgery, University Hospital, Goethe University Frankfurt, 60590 Frankfurt am Main, Germany; olivia.von.jan@gmail.com (O.V.J.); maren.janko@kgu.de (M.J.); christoph.nau@kgu.de (C.N.); marzi@trauma.uni-frankfurt.de (I.M.); d.henrich@trauma.uni-frankfurt.de (D.H.); rene.verboket@kgu.de (R.D.V.); 2 Department of Orthopedics and Traumatology, University Hospital, Johannes Gutenberg-University, 55131 Mainz, Germany; ulrike.ritz@unimedizin-mainz.de

**Keywords:** bone quality, bone healing evaluation, animal model for bone healing, bone healing score

## Abstract

(1) Background: Bone healing is a complex process that can not be replicated in its entirety in vitro. Research on bone healing still requires the animal model. The critical size femur defect (CSFD) in rats is a well-established model for fractures in humans that exceed the self-healing potential. New therapeutic approaches can be tested here in vivo. Histological, biomechanical, and radiological parameters are usually collected and interpreted. However, it is not yet clear to what extent they correlate with each other and how necessary it is to record all parameters. (2) Methods: The basis for this study was data from three animal model studies evaluating bone healing. The µCT and histological (Movat pentachrome, osteocalcin) datasets/images were reevaluated and correlation analyses were then performed. Two image processing procedures were compared in the analysis of the image data. (3) Results: There was a significant correlation between the histologically determined bone fraction (Movat pentachrome staining) and bending stiffness. Bone fraction determined by osteocalcin showed no prognostic value. (4) Conclusions: The evaluation of the image datasets using ImageJ is sufficient and simpler than the combination of both programs. Determination of the bone fraction using Movat pentachrome staining allows conclusions to be drawn about the biomechanics of the bone. A standardized procedure with the ImageJ software is recommended for determining the bone proportion.

## 1. Introduction

Bone healing, also known as fracture healing, is a complex biological process through which the body repairs and regenerates damaged bone tissue. Detailed knowledge of the biological processes is necessary for the treatment of bone defects [1,2,3]. When a bone fractures, whether due to trauma, disease, or surgery, the body initiates a series of events to restore the bone’s structural integrity. This process involves the coordination of various cells, growth factors, and signaling molecules in a well-orchestrated sequence. The initial phase of bone healing is known as the inflammatory phase, which starts immediately after the fracture occurs [4,5,6]. This phase involves the release of chemical signals and the influx of immune cells to the fracture site. Inflammatory cells, such as neutrophils and macrophages, remove debris and prepare the damaged area for subsequent healing processes [7,8]. Following the inflammatory phase, the reparative phase begins. This phase is characterized by the formation of soft callus and hard callus. Initially, soft callus forms around the fracture site, composed of a combination of blood clot, fibroblasts, and cartilage. The soft callus acts as a bridge, connecting the broken bone ends. Next, the soft callus is gradually transformed into a hard callus through a process called endochondral ossification. In this process, chondrocytes within the soft callus mature and secrete a cartilaginous matrix, which is later mineralized by osteoblasts. This mineralized matrix gradually hardens and becomes woven bone, providing initial stability to the fracture site. As the healing process continues, the hard callus undergoes remodeling. Remodeling involves the removal of excess bone tissue and the restructuring of the bone to restore its original shape and strength. The complete process of bone healing can take several weeks to months [9].

Only animal models allow researchers to investigate the intricacies of bone healing in a holistic manner [10]. They provide an opportunity to observe the entire healing process, from initial inflammation to callus formation and remodeling, on all levels [4,11,12]. But there is still no standardized procedure for the evaluation of bone defect healing. The choice of detection methods depends on the objective. For example, an osteogenic influence at the cellular level can be well detected by genetic and serological methods [13,14,15]. These phenomena can also be investigated in cell culture. Bone quality as a fusion parameter of bone mass, bone geometry, and mechanical tissue properties is so far only accessible in animal models [16]. Typically, histological, radiological, and biomechanical parameters are collected, in order to cover as many aspects of bone healing as possible on a microscopic and macroscopic level [17,18,19]. But it is not yet clear to what extent they correlate with each other. Accordingly, it is not yet clear how necessary it is to collect all parameters.

Radiological evaluation, especially, is very time-consuming and costly. Many institutes do not have their own µCT device and must fall back on histological evaluation. Therefore, it would be very interesting to see if it is possible to correlate the histologically collected parameters to the radiologically and biomechanically collected parameters. However, µCT results also have an investigator-dependent and software-dependent component. Different free software applications are available for the analysis of µCT data, e.g., InVesalius (2.5) and ImageJ (1.54f) [20,21]. InVesalius is a free software for the reconstruction of CT and MRI images. Its main functions include file analysis, volume rendering, and manual and semi-automatic image segmentation [20]. Depending on researchers’ threshold and software choices, the results can be different. A program-specific deviation in BV/TV (bone volume/total volume) values is conceivable.

Histological evaluation usually involves the evaluation of Movat-pentachrome- and osteocalcin-stained specimens [22,23,24,25]. The evaluations are usually made by means of a computer program. Although digitization has clearly promoted standardization, factors such as threshold setting are still clearly investigator-dependent.

In summary, the question now arises as to what extent radiological, histological, and mechanical markers correlate with each other in the evaluation of bone healing.

The basis for this study was data from three animal model studies evaluating bone healing. The µCT and histological datasets/images were reevaluated and correlation analyses were then performed. The experiments and studies were all performed in the Trauma Surgery Department of the University Hospital in Frankfurt, Germany. The histological preparations were examined for their bone, cartilage, and fibrous tissue content in order to determine the bone healing score. Bone volume to total volume (BV/TV) was determined using the µ-CT images. In order to determine which of the bone healing parameters in the animal model provides the most useful results, the following hypotheses needed to be tested:

1. The histological bone healing score correlates better with the biomechanical properties of the bone defect than the bone fraction in the defect.
  a. Bending stiffness is more appropriate than maximum fracture strength.
  b. Osteocalcin expression correlates with BMD.
2. The radiological and histological analyses correlate with the biomechanical properties of the bone defect.
3. Histological analysis is at least equivalent to radiological analysis in this regard.

## 2. Materials and Methods

### 2.1. Study Selection and Overview

**Figure 1:**
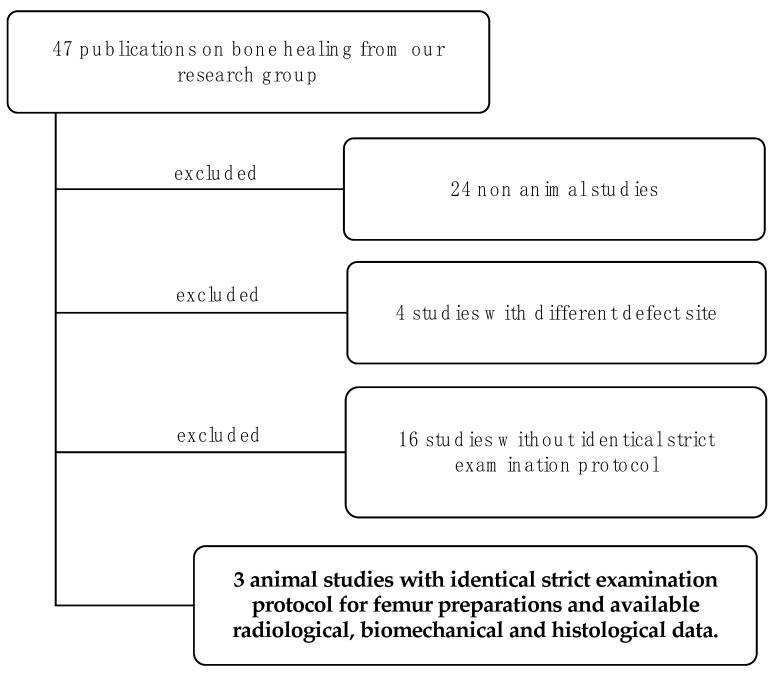
Scheme for study selection from our own publications from 2001–2023. All publications on the topic of bone healing were first reviewed for eligibility. First, all publications without animal experiments were excluded. Then, among the remaining publications, the studies on the critical size femur defect in rats were identified. Finally, three of these studies, those that used the same bone analysis for all three analysis procedures in the sequence radiology → biomechanics → histology, were selected for this retrospective study.

For this retrospective study, all publications of our research group on the topic of bone healing were first reviewed for eligibility. First, all publications without animal experiments were excluded. Then, among the remaining publications, the studies on the critical size femur defect in rats were identified. Finally, of these studies, those that used the same bone preparation for all three analysis procedures in the sequence radiology → biomechanics → histology were selected (Figure 1).

### 2.2. Post-Processing

Movat-pentachrome-stained preparations, osteocalcin-stained preparations, and µCT images from the studies already mentioned were available. These were evaluated according to a standardized scheme. In total, samples from 128 test animals could be included in the analyses. ImageJ software was used for postprocessing evaluation of the histological slides [20]. For µCT processing, the software programs CT-Analyzer (Bruker Corporation, 40 Manning Road, Billerica, MA, USA), ImageJ (https://imagej.nih.gov/ij/) 18 July 2023, and *InVesalius* 3.1 (https://invesalius.github.io/) 13 July 2023 were used [20,26,27,28,29].

#### 2.2.1. Histology

Representative Movat-pentachrome-stained sections of the defect zone were selected and processed with ImageJ. After extraction of the defect zone (polygon selections), the area was calculated. Subsequently, Color Threshold was used to mark the individual tissue types and determine their areas.

#### 2.2.2. µ-CT

From the available µ-CT datasets, the image sequences depicting the bone defect were first selected using the manufacturer’s own software (CT-Analyser, Bruker Corporation, 40 Manning Road, Billerica, MA, USA). Subsequently, the analysis was performed with the two software programs ImageJ and InVesalius [20,26].

The image sequence of the defect area, previously selected by CT-Analyser, was imported into ImageJ sorted numerically. These µ-CT images were imaged in cross-section. The bone was displayed in different shades of gray depending on the density. The “Color Threshold” was used to mark the bone outside the defect area so that the newly formed bone fraction could subsequently be determined. The threshold setting is usually in the range of 60–255, but the range may differ depending on the µCT image. No single threshold can be used for all studies because bone mineralization is not constant and bone volume varies widely between groups. The unsegmented original image was used as an aid for selecting the threshold range. In the next step, the marked image sequence was analyzed with previously defined presettings. For the total volume (TV), the area was 3.2 × 10^3^ − 6 × 10^7^ µm^2^, including the holes. To avoid artifacts, a minimum size of 3000 µm^2^ was set. The image depth in the frame was 18 µm. For the bone volume, the area range was 0–5 × 10^7^ µm^2^. The holes were not included. Subsequently, the bone volume/total volume (BV/TV) was calculated from the sums of the individual volumes.

The analysis was analogous to the previous image analysis. After extraction of the defect area, the newly formed bone was marked with a threshold in the range 72–255. In addition to coronal, axial, and sagittal section planes, a 3D reconstruction of the defect area was created. The TV and BV could be calculated directly from this. Subsequently, the BV/TV was calculated. For two-dimensional analysis of the defect area, sagittal section planes were processed using ImageJ. Bone mineral density was determined using the CT-Analyzer program.

### 2.3. Bone Healing Score

With the aid of the values of the Movat-pentachrome-stained preparations obtained by ImageJ, it was then possible to calculate the bone healing score according to Han et al. [30]. To determine the bone healing score, the proportion of newly formed bone, the proportion of cartilage, the proportion of fibrous tissue, and the remnant defect were required. The remnant defect described the area of the bone defect in which no new bone was formed. This meant that it was calculated by subtracting the newly formed bone from the total defect area. The bone healing score could be a maximum of 40 points and a minimum of 0 points. A score of 40 points indicated a high osteogenic potential and 0 points indicated a very low osteogenic potential. Newly formed bone was calculated as a proportion of the total bone defect, newly formed cartilage as a proportion of the remnant defect, fibrous tissue as a proportion of the remnant defect, and the remnant defect as a proportion of the total bone defect. Points were assigned from 0 to 10 in each case. The proportion of bone and cartilage had a positive effect and the proportion of the remnant defect and fibrous tissue had a negative effect.

### 2.4. Statistics

Statistical analyses were performed using Bias V11.12 software (Epsilon Verlag, Darmstadt, Germany). Scatterplots were used to show relationships between different parameters. For statistical analysis, the nonparametric Mann–Whitney U test was used for two comparison groups, and the Kruskal–Wallis test with Bonferroni–Holm corrected Conover–Iman post hoc analysis was used for more than two comparison groups. Statistical relationships between paired datasets were analyzed using Spearman rank correlation. Effect size, i.e., assessment of the correlation coefficient rho of the correlation, was classified into four levels (<0.2: poor—0.2–0.4: weak—0.4–0.6: moderate—0.6–0.8: strong—>0.8: optimal) according to Evans et al. [31].

For all statistical analyses, a *p* value < 0.05 was considered statistically significant, and a *p* value < 0.1 was considered a statistical trend.

## 3. Results

Three studies met the selection criteria (Table 1). All three studies were performed on the critical size femur defect in rats. The healing time was 8 weeks.

Study 1 investigated whether transfection of bone marrow mononuclear cells (BMCs) with antagoMiR-92A and antagoMiR-335 improves bone healing in the 5 mm rat critical size femur defect model (CSFD). It was observed in the group with aM-335 that there was significantly increased fracture load and callus formation compared to the scramble RNA group. In addition, significantly increased callus formation was present in the group with the combination of aM92A + aM335 compared with the scramble RNA group [32].

Study 2 investigated the optimal BMC concentration to support bone healing in the 5 mm rat CSDF model. It was shown that in the 5 mm rat femoral defect model, the effective BMC concentration was 1–5 × 10^6^ BMC/mL. Here, there was significantly increased BMD, as well as callus formation. A higher concentration led to decreased new bone formation and BMD [33].

Study 3 investigated the extent to which the process of membrane induction can be improved in the restoration of large bone defects using the Masquelet technique [12,22,34]. In the course of this, the current gold standard, i.e., filling the induced membrane with syngeneic cancellous bone, was compared with filling with granulated demineralized bone matrix (g-DBM) or fibrous demineralized bone matrix (f-DBM) with or without BMC in the 10 mm rat CSDF [4,12,35]. In summary, a heterogeneous picture emerged regarding the efficiency of f-DBM as a filler in the bone defect. Although bone maturation was locally enhanced in many other aspects, f-DBM + BMC + Masquelet technique could not provide convincing results in the treatment of large bone defects.

### 3.1. Comparison of Two- and Three-Dimensional Analysis Using InVesalius to Calculate BV/TV

There were two methods used to determine the BT/TV in this study. A representative sagittal section was used for two-dimensional analysis on the one hand, and three-dimensional defect area construction via InVesalius on the other. Both methods were compared to identify the optimal method. The correlation analysis showed a significant correlation with high effect size between both methods (rho = 0.9622 and *p* = 0.0000). Thus, both values were shown to provide equivalent results. For this reason, the simpler method using ImageJ can be used in the future.

### 3.2. Correlation Analyses

All three studies included a total of 128 different rat femora, which were examined for their biomechanical, radiological, and histological properties. Of these, at least 109 specimens could be used for the correlation analyses, depending on the analysis. The rank correlation according to Spearman with the correlation coefficient rho was used. Due to the high number of available specimens and corresponding parameters (*n* > 30), the Valz–Thompson method with two-sided approximate test with Student t-approximation or Gaussian approximation was used. Table 2 shows an overview of the correlation analysis results.

**Figure 2:**
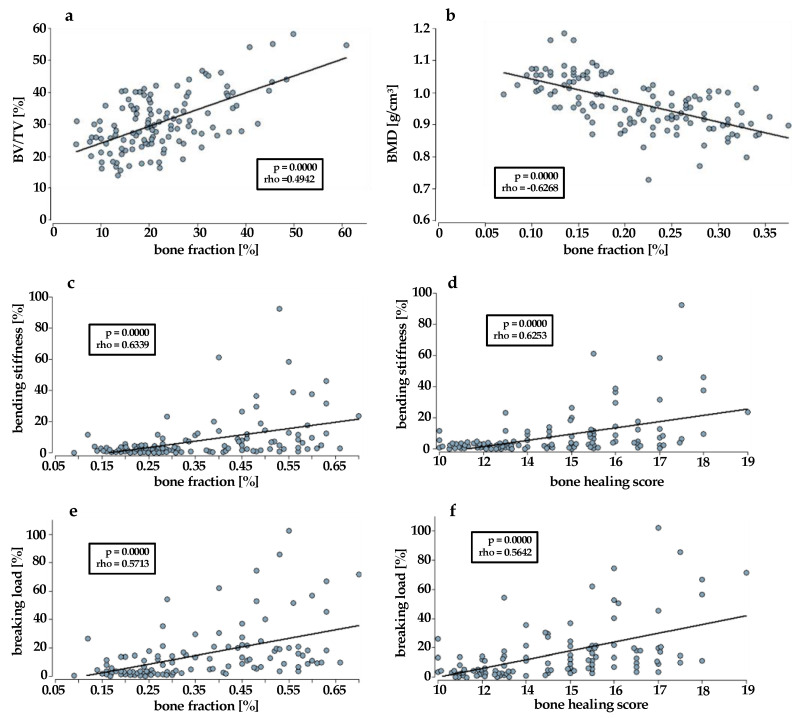
Correlations between (**a**) bone fraction (osteocalcin stain) and BV/TV (InVesalius + ImageJ), (**b**) bone fraction [%] and BMD [g/m^2^], (**c**,**d**) bone fraction (Movat pentachrome) or bone healing score and bending stiffness [%], (**e**,**f**) bone fraction (Movat pentachrome) or bone healing score and breaking strength [%]. BMD: bone mineral density; BV: bone volume; TV: total volume. *p* < 0.05 is significant.

The correlation analyses between the histological and radiological parameters yielded heterogeneous results. These varied depending on the use of BV/TV calculated with Image J or BV/TV calculated with InVesalius and ImageJ, and on the bone fraction derived from Movat pentachrome or osteocalcin staining. The correlation analysis of bone fraction (osteocalcin) and BV/TV (InVesalius + ImageJ) was highly significant (*p* = 0.0000), with a moderate effect strength (rho = 0.4942) (Figure 2a).

The other three analyses indicated only poor correlations between bone fraction and BV/TV. For bone fraction (osteocalcin stain) vs. BV/TV (ImageJ) it was rho = 0.1667 and *p* = 0.0656 (*p* > 0.05). For bone fraction (Movat pentachrome staining) vs. BV/TV (ImageJ) it was rho = −0.4907 and *p* = 0.0000, and for bone fraction (Movat pentachrome staining) and BV/TV (InVesalius + ImageJ) it was rho = −0.1207 and *p* = 0.1826.

The correlation analysis between bone fraction (osteocalcin) and BMD revealed a significant positive correlation with weak effect size (rho = 0.2941, *p* = 0.0012). Strikingly, there was a strong negative correlation between bone fraction (Movat pentachrome) and BMD (rho = −0.6268, *p* = 0.0000) (Figure 2b). This meant that the higher the BMD was, the lower the histologically assessed bone fraction was.

In addition, the correlation analysis between the bone healing score and BV/TV (InVesalius + ImageJ) showed no correlation, with rho = −0.0788 and *p* = 0.3839. When using BV/TV (ImageJ), there was a negative significant correlation with moderate effect size, with rho = −0.457 and *p* = 0.0000 (*p* < 0.05).

The correlation analysis between the bending stiffness and the bone fraction (Movat pentachrome) showed *p* = 0.0000. The correlation index was rho = 0.6339; thus, there was a significant correlation of strong effect size (Figure 2c). The same could be observed in the correlation analysis between bending stiffness and bone healing score. The correlation index was rho = 0.6253 and *p* = 0.0000. Accordingly, a significant positive correlation was observed (Figure 2d).

Similar results were obtained when analyzing the correlation between the breaking load and the bone fraction (Movat pentachrome), as well as the breaking load and the bone healing score. In each case, there was a significant positive correlation with moderate effect size. When using the bone fraction, the correlation index was rho = 0.5713 and *p* = 0.0000 (Figure 2e), and when using the bone healing score, the correlation index was rho = 0.5642 and *p* = 0.0000 (Figure 2f).

The correlation analyses between the breaking load and the bending stiffness and osteocalcin expression in the defect (bone fraction), showed only weak but significant positive correlation (rho = 0.2482, *p* = 0.0093; rho = 0.2593 and *p* = 0.0065). The amount of cartilage in the defect did not correlate with the breaking load, bending stiffness, or bone fraction. There was only a weak but significant correlation for the breaking load and the bending stiffness versus BV/TV (InVesalius + ImageJ) (rho = 0.2848, *p* = 0.0025; rho = 0.2144, *p* = 0.0238). The analysis of breaking load versus BMD (rho = −0.4164, *p* = 0.0000) and bending stiffness versus BMD (rho = −0.4762, *p* = 0.0000) showed a significant negative correlation, as did the correlation analysis for breaking load (−0.3675, *p* = 0.0001) and bending stiffness (rho = −0.4121 and *p* = 0.0000) versus BV/TV (ImageJ).

## 4. Discussion

The aim of this study was to compare and evaluate histological, radiological, and mechanical methods for determining the actual state of bone healing. Datasets of µ-CT tests, three-point bending tests (bending stiffness and breaking load), histological preparations (Movat Pentachrome and osteocalcin stains), and calculated parameters like BMD, bone percentage, and bone healing scores were correlated, taking into account different processing protocols. However, an important prerequisite for this was the collection of data under a highly standardized examination protocol using the same bone specimen for radiological, biomechanical, and histological examination. The selected studies were all performed in the same laboratory, with the same set of instruments according to an absolutely standardized protocol within a few years. This was the only way to avoid the high interspecific variability in bone healing and to perform valid correlation analyses [31]. Overall, the sequential examination of one and the same bone, in the order of radiology—biomechanics—histology, proved to be advantageous [36]. To our knowledge, this standardized procedure using only one bone and processing with a highly standardized procedure has not previously been published.

In summary, it was shown that histological and biomechanical parameters correlated with good effect strength. Here, especially the bending stiffness compared to the breaking load stands out. It showed a strong significant correlation with the bone healing score and the bone fraction (Movat pentachrome staining), in contrast to the fracture load. Bone healing score and bone proportion (Movat pentachrome staining) did not show such a clear difference. At the same time, only a very weak, non-significant correlation was present between the radiological and biomechanical parameters. The results of the correlation analyses between the histological and radiological parameters were similar, with the exception of the moderate correlation between the osteocalcin content and the BV/TV (InVesalius + ImageJ).

A review of the subject-related literature reveals that collection and analysis of radiological, histological, and biomechanical parameters of bone healing is very investigator-dependent and not standardized. In most cases, these studies are not concerned with the treatment of bone defects, but with the investigation of various influences on the bone condition. Most commonly, µ-CT, the three-point bending test, and BMD are used. In addition, osteocalcin, directly detectable in bone and serum, is a frequent additional parameter. A histological bone healing score is used in very rare cases. For the µCT analysis, the settings of the µCT are usually mentioned, but not with which program and on which key data the collected µCT images were evaluated. This makes study comparison difficult. Mainly, it was the histological and biomechanical parameters that correlated well with each other. With a higher effect size, bending strength was shown to be superior to fracture strength. Both the correlation analysis between the bending stiffness and the bone healing score, and the analysis between the bending stiffness and the bone percentage (Movat pentachrome staining), showed a positive and significant correlation of strong effect strength. In contrast to the bone fraction, the bone healing score includes the cartilage and fibrous tissue fractions. Cartilage contributes to a higher bone healing score with its high osteogenic potential but does not contribute to the bones’ stability. Fibrous tissue, as a sign of impaired bone healing, reduces the score [37,38].

However, these parameters do not seem to have a significant influence on the correlation analyses, so the more time-consuming determination of the bone healing score can be dispensed with. Critical factors such as the determination of the cartilage proportion—small changes in the color threshold in ImageJ led to strong variations with regard to the detected areas—would thus be omitted. Furthermore, the similar results between the bone fraction and the bone healing score can be explained by the large influence of the bone fraction [8]. Nevertheless, the advantage of the bone healing score is that, compared to the micro-CT, not only the bone fraction is taken into account, but a distinction is made between bone, cartilage, and fibrous tissue.

Except bone fraction (osteocalcin) vs. BV/TV (InVesalius/ImageJ) with a moderate significant correlation, there were only weak significant correlations for osteocalcin-positive bone fraction and the biomechanical or radiological results. Although previous studies have shown that osteocalcin concentration has an effect on bone mineral density (BMD), bone strength, and bone bending stiffness, the results have been inconsistent [39,40]. Osteocalcin is an important protein produced by osteoblasts and distributed in the bone cortex and trabeculae [41]. There, osteocalcin plays an important role in the early phase of bone healing and represents a major influence on the regulation of osteoblastic activity [42]. Studies have shown that osteocalcin concentration in blood is increased in osteoporosis as well as in fractures [43]. Thus, osteocalcin has been determined in the blood or urine of rats in other studies and serves as a marker of new bone formation. An increase in osteocalcin concentration in blood consistent with radiological parameters, i.e., BV/TV, was observed in various rat studies, but in most studies, correlation analyses were not performed or the result was not significant [44,45]. In this regard, it would be interesting to analyze the correlation between osteocalcin determined in blood and biomechanical, radiological, and histological parameters. Omission of osteocalcin histology by serum determination would be conceivable.

A very weak, nonsignificant correlation dominated between the radiological and biomechanical parameters. The results of the correlation analyses between the histological and radiological parameters were similar. This is surprising and contrary to expectations, since radiological examinations are commonly considered to be very solid parameters. For example, it has already been confirmed in several studies that measurements by µCT are very accurate and consistent with histologically obtained data in animals and humans [46,47,48]. However, it turns out that the collection, as well as the processing, of µ-CT datasets are performed very heterogeneously. For µCT analysis, the settings of the µCT are usually mentioned, but not the software used to analyze the collected µCT images. So, it remains difficult to compare the results between different studies. For example, the software NRecon, but also Analyze 12.0 and Tri/3D-BON software, are used in different studies, but details of the analysis procedures were not provided [49,50,51]. The problem is well known and is also addressed in the study by Bouxsein et al. [28]. However, they were mainly concerned with the settings of the µCT device, not with the different software programs and resulting deviating results. Here, a standardized procedure is urgently needed overall. Standardized µ-CT conditions and data processing would be desirable. For this purpose, at least the use of the same programs would be desirable.

In this study, therefore, two processing algorithms were investigated using two different softwares. On the one hand, a combination of InVesalius + ImageJ, and on the other hand, solely imageJ, were used for reconstruction and analysis. Here, no superiority was found for the more complex processing by means of InVesalius/ImageJ. This indicates that one method of calculating BV/TV is sufficient for the subsequent radiological analysis. However, a major factor is the threshold selection. Even in the studies examined here, an individual dataset threshold adjustment was necessary to increase segmentation accuracy. It has also been observed in other studies that a uniform threshold increases inaccuracy [46]. It is problematic when the image is analyzed with either too high or too low a threshold, and the results differ accordingly [28,46].

Another uncertain factor in the analysis of the datasets is artifact formation, in which case the image sequence section of the bones cannot be accurately represented. As a result, the corresponding sections cannot be included in the calculation of BV, TV, and BV/TV. Interestingly, no artifact formation occurred in the evaluation using the combination of InVesalius and ImageJ.

There was a negative but significant correlation between BMD and ultimate load or bending stiffness. This negative correlation was previously found by Rosales Rocabado et al. in their study of bone morphology and fracture strength of rat femora after ovariectomy [52]. In addition, correlation analyses revealed a positive correlation between BV/TV and cortical volume, and between BV/TV and cortical porosity. The study concluded that BMD is not sufficient to determine the absolute fracture risk of the bone. Fracture load may vary depending on bone location, type, and composition [52,53]. BMD describes bone mineral density, but in this process, porous trabecular bone tissue is considered as solid material due to limited image resolution [53,54].

A human study has also shown that fracture risk depends on cortical bone thickness, as well as unfavorable bone composition, and not on BMD [55]. The fracture load is influenced more by trabecular connectivity than by BMD [56]. It is difficult to make final statements about the applicability of the results shown here to human or other animal models. This requires further structured data and, in the case of human studies, the collection of histological specimens.

## 5. Conclusions

The histological bone score correlates better with the biomechanical properties of the bone defect than the bone fraction in the defect. The bending stiffness is also superior to the breaking load. Osteocalcin expression correlates significantly with BMD, but with low effect size. It is of limited use as an evaluation parameter for bone quality, but it is well suited for monitoring the development and maturation of bone tissue. The hypothesis that radiological and histological analyses correlate with the biomechanical properties of the bone defect can be refused. Histological analysis based on Movat pentachrome staining showed a close correlation with the mechanical parameters. This staining should be given more weight in the evaluation of the bone healing situation.

Finally, we recommend the examination sequence of radiology-biomechanics-histology for the same bone.

## Figures and Tables

**Table 1:** Titles of the three studies that met the selection criteria. All three studies were performed on the critical size femur defect in rats. The healing time was 8 weeks.

Study	Number of Examined Bones
Improvement of Bone Healing by Neutralization of microRNA-335-5p, but not by Neutralization of microRNA-92A in Bone Marrow Mononuclear Cells Transplanted into a large Femur Defect of the Rat [32].	20
2. Determination of the effective dose of bone marrow mononuclear cell therapy for bone healing in vivo [33].	33
3. The Induced Membrane Technique—The Filling Matters: Evaluation of Different Forms of Membrane Filling with and without BMC in Large Femoral Bone Defects in Rats [4].	75

**Table 2:** Overview table of the correlation analyses. The *p*-value (*p* < 0.05 = significant), the correlation coefficient rho, and the respective effect size are presented for each of the different analyses.

*p*-Value	rho		Effect Strength
**histological vs. radiological**			
bone fraction (Movat pentachrome) vs. BMD	0.0000	−0.6268	strong
bone fraction (osteocalcin) vs. BV/TV (InVesalius + ImageJ)	0.0000	0.4942	moderate
bone healing score vs. BV/TV (ImageJ)	0.0000	−0.4570	moderate
bone fraction (Movat pentachrome) vs. BV/TV (ImageJ)	0.0000	−0.4907	moderate
bone fraction (Movat pentachrome) vs. BV/TV (InVesalius + ImageJ)	0.1826	−0.1297	weak
bone healing score vs. BV/TV (InVesalius + ImageJ)	0.3839	−0.0788	weak
bone fraction (osteocalcin) vs. BV/TV (ImageJ)	0.0656	0.1667	weak
bone fraction (osteocalcin) vs. BMD	0.0012	0.2941	weak
**histological vs. biomechanical**			
bone fraction (Movat pentachrome) vs. bending stiffness	0.0000	0.6339	strong
bone healing score vs. bending stiffness	0.0000	0.6253	strong
bone fraction (Movat pentachrome) vs. breaking load	0.0000	0.5713	moderate
bone healing score vs. breaking load	0.0000	0.5642	moderate
bone fraction (osteocalcin) vs. breaking load	0.0093	0.2482	weak
bone fraction (osteocalcin) vs. bending stiffness	0.0065	0.2593	weak
**biomechanical vs. radiological**			
bending stiffness vs. BV/TV (ImageJ)	0.0000	−0.4121	moderate
bending stiffness vs. BMD	0.0000	−0.4762	moderate
breaking load vs. BMD	0.0000	−0.4164	moderate
bending stiffness vs. BV/TV (InVesalius + ImageJ)	0.0238	0.2144	weak
breaking load vs. BV/TV (InVesalius + ImageJ)	0.0025	0.2848	weak
breaking load vs. BV/TV (ImageJ)	0.0001	−0.3675	weak

## Data Availability

The data presented in this study are available on request from the corresponding author.
